# The buffet challenge: a behavioral assessment of eating behavior in adolescents with an eating disorder

**DOI:** 10.1186/s40337-024-00968-3

**Published:** 2024-01-18

**Authors:** Marita Cooper, Connor Mears, Kerri Heckert, Natalia Orloff, Rebecka Peebles, C. Alix Timko

**Affiliations:** 1 Department of Child and Adolescent Psychiatry and Behavioral Sciences, Children’s Hospital of Philadelphia, Philadelphia, PA USA; 2 Department of Clinical Nutrition, Children’s Hospital of Philadelphia, Philadelphia, PA USA; 3 The Craig Dalsimer Division of Adolescent Medicine, Department of Pediatrics, Perelman School of Medicine, University of Pennsylvania, Philadelphia, PA USA; 4 Department of Psychiatry, Perelman School of Medicine, University of Pennsylvania, Philadelphia, PA USA; 5 Roberts Center for Pediatric Research, 2716 South Street, Philadelphia, PA 19146 USA; 6 Present Address: Equip Health, Philadelphia, USA; 7 Present Address: Department of Psychology, West Chester University, West Chester, PA USA; 8 Present Address: Monte Nido & Affiliates, Philadelphia, USA

**Keywords:** Eating disorders, Anorexia nervosa, Adolescent, Family-based treatment

## Abstract

**Objective:**

Eating disorders are characterized by disturbances in nutritional intake and abnormal mealtime behaviors. Laboratory eating paradigms offer a unique opportunity to accurately measure dietary intake and eating behaviors, however, these studies have predominantly occurred in adults. This paper describes the development and preliminary psychometric examination of the Buffet Challenge, a laboratory-based meal task for youths with an eating disorder.

**Method:**

We recruited and assessed 56 participants as part of a randomized controlled trial of Family-Based Treatment for adolescents with anorexia nervosa. Adolescents completed the Buffet Challenge at baseline, midway through treatment (~ week 16 of a 6 months course), and end of treatment. Participants and their parents also reported eating disorder symptomatology and treatment related variables of interest were recorded.

**Results:**

All adolescents were willing to complete the Buffet Challenge at all time points, although one refused to give up their cellphone, and there were no significant adverse events recorded. Preliminary results are presented.

**Conclusions:**

Our initial pilot of this task in adolescents with anorexia nervosa demonstrates its acceptability, although investigation of our hypotheses was hindered by significant missing data due to COVID-related research shutdowns. Future studies should replicate procedures in a larger sample to ensure analyses are adequately powered.

**Supplementary Information:**

The online version contains supplementary material available at 10.1186/s40337-024-00968-3.

## Introduction

Eating disorders (ED) are characterized by disturbances in nutritional intake and abnormal mealtime behaviors [1]. For individuals with anorexia nervosa (AN) low caloric intake and disordered eating behaviors are associated with more severe psychopathology and poorer prognosis [1, 2]. These behaviors can be measured via self-report or interviews [3]; yet such approaches are limited by inaccurate reporting, inconsistent associations with actual intake, and biases of retrospective recall [4, 5]. Laboratory eating paradigms afford a rigorous approach to observe dietary intake, food choice, and mealtime behaviors [3].

The ED field has a robust history of laboratory eating paradigms in adults [3, 6], although meal content and variables collected vary. Single item or forced-choice meals [1, 7] assess intake and eating rate. Alternatively, buffet or multi-item meals where participants select from available foods including those typically avoided and “safe” food items [3, 8, 9] may better replicate naturalistic food choice. Buffet and multi-item meals can assess both nutritional data as well as aberrant mealtime behaviors [1], hunger/satiety [10, 11], psychological or neural correlates of food intake [7, 12, 13], and physiological responses to intake [14–16]. Data from laboratory meals can distinguish adults with AN from healthy controls [12], determine treatment stage [8, 15], predict long-term prognosis, or evaluate treatment outcomes [17, 18].

Normative and disordered eating behaviors differ from youth to adulthood, emphasizing the need for developmentally-appropriate adaptations in laboratory eating paradigms [19, 20]. Longitudinal data suggest children consume more fruit, dessert, dairy, and mixed meat than adults; whereas, adults are more likely to report consuming poultry, cheese, seafood, and salty snacks [21]. These differences may relate to changing taste preferences, i.e., children/adolescents show greater preference toward sweet foods and beverages than adults [22] as well as influences of increasing food autonomy and lifestyle changes [23]. Eating behavior/expectations during laboratory meals may also differ from adults to adolescents with AN; given that first line treatment during childhood/adolescence is family-based treatment, where caregivers serve and supervise meals [24, 25].

All laboratory meal paradigms in youth with an ED, to date, have focused on binge episodes and loss-of-control eating and have contributed to defining overeating episodes in youth [26], examining associations between intake and mood [27], and exploring psychophysiological correlates [28]. These paradigms are specifically tailored to induce binge episodes, including a focus on highly palatable foods. Although youth with restrictive ED also engage in binge eating behavior [29], neither the procedures nor variables collected in these paradigms were developed to assess restrictive eating patterns such as avoidance of calorie dense foods, low dietary variety, or mealtime ED behaviors [1, 9].

Herein we describe the development, feasibility, and preliminary psychometric examination of the Buffet Challenge, a laboratory meal paradigm for youths with AN. We conceptualize the Buffet Challenge as a behavioral assessment of flexibility in eating (including approaching the buffet and serving a wide variety of food). Our sample comprises adolescents with AN who underwent treatment in a randomized clinical trial [30]. We operationalized disordered buffet consumption as the following: lower caloric intake, caloric density, and intake from fats, and higher non-caloric liquid intake. We hypothesized that disordered buffet consumption would be associated with higher AN psychopathology. Secondly, we hypothesized that less disordered buffet consumption at mid-treatment and end-of-treatment (EOT) would be seen in participants classified as partially or fully remitted compared to those categorized as not remitted as well as those undergoing later phases of FBT, when compared to those in treatment Phase I. Finally, we predicted that lower baseline disordered buffet consumption would predict early treatment response and lower EOT disordered buffet consumption would predict post-treatment outcome.

## Methods

### Participants

We recruited 59 participants as part of a randomized controlled trial (see Timko et al. [30]). Three were removed who did not complete the Buffet Challenge at any time point (one individual was Kosher and two participants completed all time points virtually due to COVID-related shutdowns), resulting in 56 adolescents (48 cisgender females, 8 cisgender males) with AN (four with binge/purge subtype and 52 with restricting subtype). The sample were aged 15.3 years (*SD* = 1.6), with a BMI z-score of − 0.68 (see Table 1 for descriptive and clinical data). The sample were mostly White (*n* = 52, 92.9%), with the remainder identifying as Asian (*n* = 2, 3.6%), Black (*n* = 1, 1.8%), or "Other” (*n* = 1,1.8%). One adolescent identified as Hispanic/Latina (1.8%). Participants were randomized to one of three conditions—Family Based Treatment (*n* = 20), FBT + Parent-focused Cognitive Remediation Therapy (*n* = 18), or FBT + Adolescent-focused CRT (*n* = 18).

**Table 1:** Clinical descriptive and eating disorder psychopathology data on sample

	Baseline (*N* = 52) M (SD)	Mid-treatment (*N* = 31) M (SD)	End of Treatment (*N* = 21) M (SD)
Age (yrs)	15.4 (1.6)	15.8 (1.6)	15.9 (1.8)
BMI z-score	− 0.7 (0.8)	0.2 (0.6)	0.2 (0.6)
Recommended daily energy intake (kcal)	4140.6 (504.9)	4492.3 (651.9)	4308. 8 (208.9)
EDE-Q global	2.7 (1.7)	1.3 (1.5)	1.2 (1.4)
ABOS (mother report)	26.8 (9.3)	17.3 (9.0)	9.7 (7.6)
ABOS (father report)	24.9 (9.8)	15.2 (9.6)	11.3 (8.2)

#### Impact of COVID-19

Due to the COVID-19 pandemic, we halted in-person research in March 2020 and adapted study protocols to ensure participant/researcher safety. Subsequently, four participants did not complete the Buffet Challenge at baseline, with 14 and 19 unable to complete the Buffet Challenge at mid-treatment and EOT, respectively. In addition to COVID-related disruptions, 11 participants dropped out of the study by mid-treatment and five did not complete EOT assessments (total *n* = 16). This resulted in 21% (*n* = 12) of participants completing all time points, with 43% (*n* = 24) of participants completing two time points, and 36% (*n* = 20) completing only one buffet. We compared these three groups using independent samples t-tests, observing no significant differences among adolescents who completed all three Buffet Challenges and those who only completed one or two time points on age, BMI z-score, or caloric consumption (including proteins, fats, or carbohydrates) at any time point (*p*s < 0.05). We did not control for this in further analyses. The final sample (*N* = 56) included 52 participants at baseline, 31 at mid-treatment, and 21 at EOT.

### Development of the buffet challenge

To foster reproducibility of the Buffet Challenge, we provide full details regarding: (1) selection of food items and layout; (2) accommodations for dietary requirements; and (3) task administration in the Additional file 1.

### Measures

#### Eating disorder examination-questionnaire (EDE-Q) [31]

The EDE-Q is a self-report measure assessing ED psychopathology over the past 28 days. The instrument comprises four subscales averaged to generate a global score. We found excellent internal consistency of the EDE-Q global score, with McDonald’s ω = 0.97 at baseline.

#### Anorectic behavior observation scale (ABOS) [32]

The ABOS is a 30-item collateral-report questionnaire assessing eating and exercise behavior during the prior month. Higher scores on the ABOS represent greater AN behavior. At baseline, we found acceptable internal consistency (maternal McDonald’s ω = 0.70; paternal McDonald’s ω = 0.86) of the ABOS.

### Procedure

Adolescents completed the Buffet Challenge at baseline, midway through treatment, and EOT. The mid-treatment assessment occurred ~ week 16/post-session 11 when it was expected that adolescents would transition into Phase II of FBT [24, 30], which focusses on shifting toward age-appropriate involvement in food-related decision-making. All assessments were monitored via closed circuit video in real time to monitor adolescent safety (e.g., choking, panic attack, self-harm) and intervene as necessary. We recorded all assessments and two raters independently coded observable behavior. Individualized calorie and weight goals were calculated via standard program procedures [33]

### Scoring the buffet challenge

We assessed amount of food served (i.e., pre-buffet food item minus weight of food left on serving table) and consumed (i.e., pre-buffet food weight minus weight of food served and food remaining) for each food item. Caloric and macronutrient intake of total food served and total food consumed were calculated. We also calculated the following variables: percentage of daily intake consumed, caloric density (i.e., total kcal consumed/total grams consumed), percentage of intake from each macronutrient, and non-caloric liquid intake. Percentage of daily intake consumed was calculated as total kcal consumed during the Buffet Challenge/total recommended kcal × 100. The macronutrient breakdown was calculated as total kcal from each macronutrient consumed/total kcal consumed × 100. Finally, non-caloric liquid intake was the combined fluid ounces consumed from non-caloric liquids (e.g., water, Diet Coke).

We also recorded eating-related behaviors (see Additional file 1: Table S2). We developed these items based on observable behaviors (e.g., duration of time spent eating, frequency of label checking) including those identified by Gianni et al. [1], such as staring at food, tearing food, nibbling or picking, dissection of food, and hand fidgeting.

### Statistical analyses

Data were analyzed in IBM SPSS Statistics. Given missing data and no significant differences in outcome across treatment, we collapsed data across condition. All analyses used the following variables: percentage of daily intake consumed, caloric density, percentage of intake from each macronutrient, and non-caloric liquid intake (as defined earlier). Analyses were grounded in traditional null hypothesis testing; however, given documented concerns with interpretation of results, particularly when there is missing data [34, 35] we focused our evaluation on effect sizes and confidence intervals to examine task validity.

Our first hypothesis examined concurrent validity of the Buffet Challenge via Pearson’s/Spearman’s correlations to probe associations between buffet variables and parent-report ABOS scores at each timepoint. Our second hypothesis examined known-group validity, comparing buffet variables across remission status using Kruskal–Wallis tests at mid-treatment and EOT. Remission criteria included weight > 95% target body weight (as determined by historical growth curve) [33, 36], and self-reported ED psychopathology (recovered for boys EDE-Q < 0.61, girls EDE-Q < 1.84) [37]. Our third hypothesis compared buffet variables at mid-treatment and EOT across individuals in different phases of FBT using Kruskal–Wallis tests. We used independent t-tests/Mann Whitney U tests to examine the predictive validity of buffet intake variables. We probed differences in baseline buffet data for those who did, versus did not, attain early weight gain (i.e., 4lbs after 4 weeks of treatment) and EOT buffet intake data for those who were referred to a higher level of care in the 12 months post-treatment, versus those who were not. Referral data were abstracted from participant’s electronic health record, note that we did not have adequate data on three individuals. We decided to present all behavioral data, rather than analyzing these variables, as we did not have specific hypotheses and high missing data.

## Results

### Feasibility

All adolescents, except one youth who participated but refused to give up their phone, were willing to complete the Buffet Challenge at all time points (defined as remaining in the buffet room for 30 min without their cell phone). Other challenges during administration (104 total buffets) included unavailability of specific foods (occurrences = 13), weighing error (occurrence = 2), item taken/unable to be weighed (occurrence = 2), recording error (occurrence = 1), adolescent ate prior (occurrence = 1), vegetarian food ordered in error (occurrence = 1), and standard buffet ordered instead of vegetarian (occurrence = 1). Twelve adolescents did not consume anything during the buffet (*n* = 6 at T1, *n* = 5 at T2, and *n* = 1 at T3); all were supplemented by parents following the task. One adolescent exhibited significant distress following the Buffet Challenge at baseline but willingly engaged in the Buffet Challenge at both future timepoints. One adolescent scraped the inside of their wrist with a plastic knife during the task (the knife was removed). An on-site psychologist met with both adolescents. The first was able to engage in coping behavior and had reduced distress. The second reported that they were attempting to “get out” of treatment and subsequently agreed to continue the assessment.

### Descriptive statistics

At mid-treatment, participants completing the Buffet Challenge were in Phase I (48%) or II (45%) of FBT, with 7% in Phase III. By EOT, most participants were in Phase II (57%) of FBT, with 14% in Phase I, and 29% in Phase III. Table 2 presents macronutrient and liquid consumption, with frequency and duration of eating-related behaviors shown in Table 3. The most common ED behaviors across all timepoints were staring at food, fidgeting, and inappropriate napkin use.

**Table 2:** Descriptive amounts of caloric consumption at each time point

	Baseline (*N* = 52) M (SD)	Mid-treatment (*N* = 31) M (SD)	End of treatment (*N* = 21) M (SD)
% of participants consuming any calories	46 (88%)	26 (84%)	20 (95%)
Total food consumed (grams)	390.6 (274.2)	269.7 (202.4)	291.8 (140.1)
Total food consumed (kcals)	639.3 (399.8)^a^	530.9 (314.2)^b^	454.9 (232.1)^c^
% daily recommended intake	16.0 (9.3)^a^	12.5 (7.0)^b^	11.5 (5.7)^c^
Protein consumed (grams)	28.2 (28.5)	22.1 (22.8)	21.5 (14.1)
Protein consumed (kcal)	127.6 (113.2)^a^	105.4 (90.3)^b^	90.4 (54.0)^c^
% of intake from protein	18.3 (9.6)^a^	2123 (15.4)^b^	19.5 (9.5)^c^
Fats consumed (grams)	24.7 (24.3)	19.4 (16.8)	18.6 (12.8)
Fats consumed (kcal)	251.2 (219.8)^a^	208.6 (142.4)^b^	175.6 (111.6)^c^
% of intake from fats	33.7 (12.7)^a^	32.4 (123)^b^	34.4 (11.5)^c^
Carbohydrates consumed (grams)	68.4 (49.3)	52.9 (42.1)	56.1 (34.2)
Carbohydrates consumed (kcal)	309.4 (181.1)^a^	252.3 (153.1)^b^	236.0 (129.8)^c^
% of intake from carbs	47.9 (15.2)^a^	46.4 (17.2)^b^	46.1 (11.8)
Liquid calories consumed (kcal)	32.4 (67.0)^a^	28.0 (55.2)^b^	38.3 (59.1)^c^
Non-caloric liquid consumed (flOz)	6.5 (7.0)	8.1 (7.4)	7.4 (6.7)
Caloric density (kcal/gm)	1.3 (0.7)	1.4 (0.8)	1.4 (0.6)

^a^For BASELINE amount consumed means and standard deviations *N* = 46

^b^For MID-TREATMENT amount consumed means and standard deviations* N* = 26

^c^For END OF TREATMENT amount consumed means and standard deviations* N* = 20

**Table 3:** Frequency and duration of mealtime behaviors at each time point

	Baseline (*N* = 51)* M (SD)	Mid-treatment (*N* = 29)* M (SD)	End of Treatment (*N* = 20)* M (SD)
*Behavior latency (in seconds)*			
Approach serving table	18.7 (43.9) *N* = 49	26.5 (65.7) *N* = 27	57.1 (206.1) *N* = 20
Eating	188.0 (118.8) *N* = 43	171.8 (110.1) *N* = 24	128.6 (65.6) *N* = 19
Nibbling/picking	622.3 (375.2) *N* = 16	403.9 (267.3) *N* = 10	407.0 (403.2) *N* = 5
Serve food	39.8 (43.9) *N* = 43	82.0 (210.8) *N* = 26	77.0 (203.3) *N* = 20
*Behavior frequency*			
Approach serving table	2.4 (1.9) *N* = 45 (88%)	2.1 (1.6) *N* = 26 (90%)	1.7 (1.0) *N* = 20 (100%)
Dissecting	4.6 (4.4) *N* = 9 (18%)	2.9 (3.1) *N* = 9 (31%)	1.5 (0.8) *N* = 6 (30%)
Dropping food	2.0 (1.0) *N* = 3 (6%)	1.0 *N* = 1 (3%)	*N* = 0 (0%)
Eating whilst serving	4.5 (4.3) *N* = 8 (16%)	2.3 (2.3) *N* = 6 (21%)	5.0 *N* = 1 (5%)
Hand fidgeting	7.1 (7.1) *N* = 41 (80%)	8.3 (6.4) *N* = 22 (85%)	10.5 (7.6) *N* = 12 (60%)
Inappropriate utensil use	13.7 (23.2) *N* = 9 (18%)	9.5 (8.6) *N* = 4 (14%)	8.5 (3.5) *N* = 2 (10%)
Label checking	4.4 (8.2) *N* = 26 (51%)	3.1 (2.9) *N* = 13 (45%)	4.0 *N* = 1 (5%)
Napkin use	9.4 (7.2) *N* = 34 (67%)	8.1 (5.9) *N* = 21 (72%)	11.7 (10.5) *N* = 14 (70%)
Nibbling/picking	6.9 (6.1) *N* = 16 (31%)	10.6 (11.8) *N* = 10 (34%)	10.4 (11.3) *N* = 5 (25%)
Smearing	4.0 *N* = 1 (2%)	2.6 (1.8) *N* = 5 (17%)	*N* = 0 (0%)
Staring at food	7.7 (5.1) *N* = 47 (92%)	6.4 (5.9) *N* = 19 (66%)	6.0 (3.6) *N* = 13 (65%)
Tearing	2.7 (2.1) *N* = 15 (29%)	10.0 (10.6) *N* = 11 (38%)	3.8 (2.6) *N* = 4 (20%)
*Behavior duration (in seconds)*			
Eating	1163.4 (461.2) *N* = 44	994.7 (457.3) *N* = 23	1044.4 (406.5) *N* = 19
Serving	134.9 (94.0) *N* = 43	95.3 (93.1) *N* = 27	72.7 (65.4) *N* = 20
Staring at food	167.9 (124.7) *N* = 47	152.2 (140.9) *N* = 19	224.5 (246.4) *N* = 13

*One video at baseline and end of treatment as well as two videos at mid-treatment were not coded due to issues with video recording. The number of participants in each variable differs due to some participants completing a behavior, e.g., approaching or not approaching buffet, and is listed for each variable and timepoint

### Concurrent validity

Associations among buffet related consumption and parent-reported ED behavior differed across maternal and paternal report (Table 5). Higher maternal ABOS scores at baseline were associated with lower caloric density consumed during the Buffet Challenge. Surprisingly, higher maternal ABOS scores at EOT were associated with a significantly greater portion fats at the EOT Buffet Challenge. We did not observe significant associations between paternal ABOS scores and buffet data at any timepoint.

### Known-group validity

Results assessing buffet variables across remission status (i.e., not remitted, partially remitted, and fully remitted) are shown in Table 4. Mid-treatment Buffet Challenge data were collected for 31 adolescents (*N*_full remission_ = 15, *N*_partial remission_ = 13, *N*_not remitted_ = 3). Kruskall-Wallis tests yielded a moderate, non-significant effect across groups in caloric density consumed (see Table 4), where caloric density was lowest in the not remitted group (*m* = 0.9, *sd* = 0.3), compared to those partially (*m* = 1.1, *sd* = 0.9) or fully remitted (*m* = 1.6, *sd* = 0.7) (Table 5).

**Table 4:** Between-group differences on buffet related variables at mid-treatment and end of treatment

Variable	Groups	Variable	Mid-treatment	End of treatment
Remission status	Not remitted Partially remitted Full remission	% Daily kcal consumed	*H*(2) = 1.966, *p* = .374, η^2^ = − .001, 90% CI[− .06, .26]	*H*(2) = 2.095, *p* = .351, η^2^ = .005, 90% CI [− .07, .46]
		Caloric density	*H*(2) = 5.119, *p* = .077, η^2^ = .111, 90% CI[− .01, .35]	*H*(2) = 4.960, *p* = .084, η^2^ = .164, 90% CI[− .03, .56]
		% Intake from protein	*H*(2) = 0.438, *p* = .803, η^2^ = − .056, 90% CI[− .07, .19]	*H*(2) = 2.434, *p* = .296, η^2^ = .024, 90%CI[− .05, .34]
		% Intake from fats	*H*(2) = 1.283, *p* = .527, η^2^ = -.026, 90%CI[-.06, .21]	*H*(2) = 2.451, *p* = .294, η^2^ = .025, 90%CI[-.05, .39]
		% Intake from carbohydrates	*H*(2) = 1.971, *p* = .373, η^2^ = − .001, 90% CI[− .06, .26]	*H*(2) = 1.693, *p* = .429, η^2^ = − .017, 90% CI[− .08, .29]
		Non-caloric liquid consumption	*H*(2) = 0.781, *p* = .677, η^2^ = − .044, 90% CI[− .07, .19]	*H*(2) = 3.379, *p* = .185, η^2^ = − .077, 90% CI[− .05, .4]
FBT Phase	Phase I Phase II Phase III	% Daily kcal consumed	*H*(2) = 5.399, *p* = .067, η^2^ = .121 90% CI[− .02, .35]	*H*(2) = 1.234, *p* = .540, η^2^ = − .043, 90% CI[− .1, .23]
		Caloric density	*H*(2) = 5.999, *p* = .050, η^2^ = .143, 90% CI[− .000, .39]	*H*(2) = 1.407, *p* = .495, η^2^ = − .033, 90% CI[− .1, .34]
		% Intake from protein	*H*(2) = 0.794, *p* = .672, η^2^ = − .043, 90% CI[− .07, .16]	*H*(2) = 1.273, *p* = .529, η^2^ = − .040, 90% CI[− .1, .35]
		% Intake from fats	*H*(2) = 4.724, *p* = .094, η^2^ = .097, 90% CI[− .03, .36]	*H*(2) = 0.606, *p* = .739, η^2^ = − .077, 90% CI[− .1, .35]
		% Intake from carbohydrates	*H*(2) = 0.052, *p* = .974, η^2^ = − .070, 90% CI[− .07, .09]	*H*(2) = 0.890, *p* = .641, η^2^ = − .062, 90% CI[− .1, .33]
		Non-caloric liquid consumption	*H(2)* = 1.934, *p* = .380, η^2^ = − .002, 90% CI[− .04, .19]	*H*(2) = 1.234, *p* = .540, η^2^ = − .105, 90% CI[− .11, .23]
Higher level of care referral	No Yes	% Daily kcal consumed	*–*	*t*(16) = 0.075, *p* = .941, Hedge’s *g* = 0.037, 95% CI[− 0.945, 1.1019]
		Caloric density	*–*	(*t*(16) = 0.253, *p* = .803, Hedge’s *g* = 0.127, 95% CI[− 0.858, 1.108]
		% Intake from protein	*–*	*t*(16) = − 1.536, *p* = .144, Hedge’s *g* = − 0.770, 95% CI[− 1.776, 0.259]
		% Intake from fats	*–*	*t*(16) = − 1.578, *p* = .134, Hedge’s *g* = − 0.791, 95% CI[− 1.798, 0.240]
		% Intake from carbohydrates	*–*	Mann Whitney U = 76.000, *z* = − 0.212, *p* = .856
		Non-caloric liquid consumption	–	*t*(16) = 0.076, *p* = .941, Hedge’s *g* = 0.038, 95% CI[− 0.945, 1.020]

**Table 5:** Concurrent validity of buffet variables with eating disorder psychopathology per maternal and paternal report

Time point	Variable	BASELINE ABOS		MID-TREATMENT ABOS		END OF TREATMENT ABOS	
		Maternal	Paternal	Maternal	Paternal	Maternal	Paternal
Baseline	% Daily recommended intake	− .137	− .107	–	–	–	–
	Caloric density	− .290*	− .139	–	–	–	–
	% Intake from protein	− .009	.036	–	–	–	–
	% Intake from fats	− .094	− .062	–	–	–	–
	% Intake from carbohydrates	− .031	− .197	–	–	–	–
	Non-caloric liquid consumption	− .006	.177	–	–	–	–
Mid-treatment	% Daily recommended intake	–	–	− .276	− .143	–	–
	Caloric density	–	–	− .212	− .236	–	–
	% Intake from protein	–	–	− .032	.131	–	–
	% Intake from fats	–	–	− .215	− .224	–	–
	% Intake from carbohydrates	–	–	− .134	− .136	–	–
	Non-caloric liquid consumption	–	–	− .105	.037	–	–
End of treatment	% Daily recommended intake	–	–	–	–	.310	− .224
	Caloric density	–	–	–	–	.142	− .025
	% Intake from protein	–	–	–	–	− .089	− .260
	% Intake from fats	–	–	–	–	.738**	.167
	% Intake from carbohydrates	–	–	–	–	− .278	− .311
	Non-caloric liquid consumption	–	–	–	–	.066	− .022

*p < .05, **p < .01

For the EOT Buffet Challenge (*N* = 21), 13 adolescents met criteria for full remission, 6 for partial remission, and 2 were not remitted. Kruskal–Wallis tests revealed a large, non-significant effect across remission groups for caloric density (see Table 4), which was lowest in the not remitted group (*m* = 0.9, *sd* = 0.2), compared to those partially (*m* = 1.3, *sd* = 1.0), or fully remitted (*m* = 1.6, *sd* = 0.4). There was a medium, non-significant effect for non-caloric liquid consumption, which was highest in the not remitted group (*m* = 17.0, *sd* = 0.0), compared to those partially (*m* = 6.0, *sd* = 7.1), or fully remitted (*m* = 6.6, *sd* = 6.1).

### Associations with treatment-related variables

Results assessing buffet variables across FBT phase (Phase I, II, and II) are shown in Table 4. At mid-treatment, we found a large, non-significant effect across FBT phase in caloric density (Table 4). Participants in Phase I consumed the lowest caloric density (*m* = 1.1, *sd* = 0.7), compared with those in Phase II and III, respectively (*m* = 1.5, *sd* = 0.8; *m* = 2.4, *sd* = 0.4). Medium, non-significant effects were observed for percent of daily intake consumed, fat intake, and carbohydrate intake. Individuals in Phase I consumed approximately 8.1% (*sd* = 7.5) of their daily intake, compared with 11.4% (*sd* = 7.6) and 22.0% (*sd* = 2.5) in Phase II and III, respectively. Those in Phase I had the lowest intake from fats (*m* = 22.1%, *sd* = 15.3), compared with 30.6% (*sd* = 17.3) and 41.1% (*sd* = 5.3) in Phase II and III respectively. Percent intake from carbohydrates was lowest for those in Phase I of FBT (*m* = 37.9%, *sd* = 26.2), compared with 39.2% (*sd* = 22.8) and 45.1% (*sd* = 5.4) in Phase II and III, respectively.

At EOT, medium, non-significant effects across Phase for percentage of intake from fats (see Table 4) were observed. Fat intake was lower for those in Phase I (*m* = 31.5%, *sd* = 16.0) and Phase II (*m* = 31.2%, *sd* = 14.4), compared to those in Phase III (*m* = 36.6%, *sd* = 12.1). For percent intake from carbohydrates, intake was highest for those in Phase I (*m* = 49.1%, *sd* = 18.6) compared to those in Phase II (*m* = 40.2%, *sd* = 16.7) and Phase III (*m* = 48.7%, *sd* = 10.2). For non-caloric liquid consumption, those in Phase I consumed an average of 8.3 floz (*sd* = 8.5), compared with 7.1 (*sd* = 6.9) and 7.6 (*sd* = 6.7) in Phase II and III, respectively.

### Predictive validity

We observed only small (caloric density and non-caloric liquid intake) and negligible non-significant effects at baseline across all variables for those who did and did not achieve early weight gain (i.e., 4lbs in the first four weeks).

We compared EOT buffet data of participants who went on to a higher level of care *(N* = 5) post-treatment compared to those who did not (*N* = 13). We observed medium, non-significant effect sizes between groups for percentage of intake from fats (Table 4), where those who were referred to a higher level of care consumed on average of 22.8% (*sd* = 16.1) of their meal from fats compared to an average of 33.2% for those who were not (*sd* = 11.0). For percentage of intake from protein, those referred to a higher level of care consumed on average of 13.3% (*sd* = 12.8) of their meal from proteins compared to an average of 21.6% for those who were not (*sd* = 9.2).

## Discussion

The present study described the development and preliminary examination of the Buffet Challenge, a laboratory-based meal paradigm for youth with an ED. Pilot data demonstrates its acceptability, considering all participants were willing to participate in the task (except one adolescent unwilling to give up their phone). The most common administration challenge was availability of buffet foods; however, much of the study took place during COVID-related shutdowns and most unavailable food only happened one time, suggesting the task is feasible for future research. Future work may want to have a selection of alternate foods comparable in type and (e.g., cookies) and macronutrient breakdown.

Investigation of our hypotheses was hindered by significant missing data due to COVID-related shutdowns. Maternal-report of ED behavior at baseline was significantly associated with less calorically dense consumption, whereas there were no significant correlations between buffet variables and paternal-report at any timepoint. The relationship between maternal report of ED behavior and buffet variables at EOT was more difficult to interpret and need to be replicated. Caloric density stood out as a potential marker of recovery, demonstrating medium-large effects, albeit non-significant, across remission groups and FBT phase at mid-treatment and EOT. While results were non-significant, there was a medium effect at EOT intake from fats and protein across those were referred to a higher level of care, versus those who were not.

There were medium-large non-significant effects for percent of daily intake consumed, caloric density, macronutrient breakdown of meal, and non-caloric liquid consumption with remission status, FBT Phase, and higher level of care referral. These results appear consistent with meal-based and self-report data from prior studies in adults with AN [2, 8, 15], which highlight caloric density as both a measure of recovery and marker of prognosis. This is unsurprising considering avoidance of dietary fats and a diet low in caloric density are hallmark characteristics of AN [9]. Despite this, average macronutrient consumption of participants at all three timepoints (protein, 18–22%, fats 32–35%, and carbohydrates 46–49%) were more aligned with typical treatment recommendations (protein, 15–20%, fats 25–35%, and carbohydrates 50–60%) [38, 39] than in prior studies of adults with AN, where average fat intake represents between 20 and 25% of the meal [9, 13]. Considering that AN typically onsets in adolescence, these results may suggest that a diet low in dietary fats could contribute to the maintenance and chronicity of AN. Our findings emphasize the importance of consuming a diet high in caloric dense foods to protect against relapse following specialist care for AN.

Contrary to hypotheses, baseline buffet variables did not predict early weight gain in our sample. One possible explanation is that FBT charges parents with renourishment, such as supervising, preparing, and portioning all meals in Phase I [24]. Thus, early weight gain may reflect parental efforts in facilitating renourishment, rather than an adolescent’s capacity to select and consume adequate nutrition. This is supported by research suggesting that parental, not adolescent, factors (e.g., self-efficacy and therapeutic alliance), predicted early response to treatment in youth undergoing FBT [40, 41]. These results further emphasize the critical role of parents in recovery.

Study strengths include adapting existing meal-based tasks to reflect normative eating in adolescents, the range of variables collected, and thorough description of Buffet Challenge administration. The latter is necessary to facilitate the reproducibility of findings and rigor of research into ED behaviors in adolescents. Further, this is the first study to examine non-caloric liquid consumption and caloric intake *relative to* recommended daily caloric intake. Considering these were both related to variables of interest (FBT phase and remission status), we encourage future researchers to investigate these as markers of recovery from AN. Adolescents with AN differ critically from adults; both in terms of first line treatment [42], and normative eating behaviors (i.e., role in food preparation, dietary variety, caloric requirements) [19, 20]. We need data from meal-based tasks, such as the Buffet Challenge, that reflect real-world eating for adolescents to determine whether observations in adults with AN hold true for youth.

Despite these strengths, the Buffet Challenge was conducted in an artificial laboratory environment; future researchers should consider how to measure other realistic aspects of eating behavior such as social eating. Foods were representative of a diet typical of White American adolescents that may not reflect the diverse cuisines of youth from other cultures. Future iterations should adapt available foods to better represent the premorbid intake of youth from culturally diverse backgrounds. One final major limitation was the large amount of missing data. This severely limited our power to conduct analyses, for which we would have ideally been able to reduce alpha to control for multiple comparisons. The presence of medium to large effect sizes indicates that replication in a larger sample with more complete data is necessary. We plan to replicate and extend our findings in future studies to better understand associations between meal-based intake, AN psychopathology, remission, and treatment outcome.

In sum, the Buffet Challenge is feasible to administer and acceptable to adolescents with AN. Preliminary results suggest that caloric density of intake may be associated with current and future markers of remission. Future studies should replicate procedures in a larger sample to ensure analyses are adequately powered.

### Supplementary Information

**Additional file 1**. Supplemental information.

## Data Availability

The data that support the findings of this study are available on request from the corresponding author.
