# Supplementary material for: The buffet challenge: a behavioral assessment of eating behavior in adolescents with an eating disorder
Source: J Eat Disord. 2024 Jan 18;12:8. doi: 10.1186/s40337-024-00968-3 (PMC10797715; doi:10.1186/s40337-024-00968-3)

**Supplemental information**

***Selection of Food Items and Layout.*** We developed the Buffet Challenge to objectively assess food choice and eating/eating-related behaviors in adolescents with an eating disorder. Given this purpose, we chose foods with a wide variety of caloric and macronutrient compositions (see Supplemental Table 1). We initially developed a list of potential food items from those included in the Food Choice Task^43^, a computer based assessment of food choice for individuals with AN. Additional items were generated from clinical expertise of the study team members (CAT, KH, NCO), each of whom have extensive expertise in the assessment and treatment of adolescents with an eating disorder. While there was overlap between items generated by our team and those used by Sysko and colleagues (2018), we wanted to specifically include food items representative of dietary options adolescents commonly encounter in daily life, ranging from “safe” foods (e.g., carrot and celery sticks, blueberries, diet soda) to “challenge” foods (e.g., cheese pizza, brownies, chicken fingers). The full list of food items provided, serving sizes, and nutritional information are outlined in Supplemental Table 1. The study dietitian (KH), in consultation with healthy adolescent volunteers, constructed the layout of the Buffet Challenge (as seen in Figure 1).

***Dietary Accommodations.*** Food items in the Buffet Challenge were based upon a typical American omnivore diet; thus, we made some accommodations to the food list for those with dietary limitations, specifically celiac disease or vegetarianism diets. As adoption of a gluten-free or vegetarian diet could be part of the eating disorder ^44^, we only modified food items in the Buffet Challenge when: 1) family reported long-standing family or religious practice of vegetarianism; 2) documented allergy; or 3) autoimmune condition requiring avoidance of gluten. In these instances, we selected individual food item substitutions that best matched standard buffet items (e.g., mozzarella sticks instead of chicken fingers). A full list of recommended substitutions is available upon request.

***Administration of the Buffet Challenge.*** Research staff scheduled the Buffet Challenge between 11:00 am and 2:00 pm, whenever possible, and adolescents/families were instructed prior to arrival to be prepared to have lunch at the study visit. Prior to an adolescent’s arrival research staff weighed all food items using a Tanita KD-200-110 digital scale. Food items were placed on the buffet table in a standard layout (see Supplemental Figure 1). Upon entering the buffet room, a study team member reviewed all food items present with the adolescent and answered any questions they had about the items or ingredients. Adolescents were informed that no electronic devices were permitted in the room during the meal. Adolescents were then instructed to *“serve yourself a meal that you need to eat to recover from your eating disorder.”* These instructions were designed to be consistent with those provided to parents in FBT prior to the family meal ^24^. Adolescents were encouraged to return to the buffet as many times as they wished and to leave all items out after completion. Finally, adolescents were informed that they would have 30 minutes to compete the meal. This time limit was selected to reflect the typical duration of meals in our Nutritional Rehabilitation Pathway (see Peebles and colleagues^33^). The average time for a family meal in households without a child with an eating disorder ranges from 15 to 29 minutes ^45^; thus, we would expect remitted adolescents to be able to serve themselves and complete a meal in this amount of time. After providing instructions and answering any questions, the study team member left the adolescent alone in the room with the buffet.

Following the 30-minute period, the team member returned to the room. If the adolescent was still eating at this point, they were informed of the time and provided an additional 5 minutes to complete the meal. Otherwise, adolescents left the room and all food items (both those on the dining table and buffet area) were separately re-weighed to calculate amounts of each food item served and consumed. Given that all participants were either beginning FBT (baseline) or actively engaged in FBT concurrent to the assessment, immediate feedback was provided to parents following the task regarding how much the adolescent had consumed so that parents could modify the day’s intake accordingly.

Supplemental Table 1. *Buffet Challenge Food Items*

| **Food Item** | **Portion Provided to Adolescent** | **Reference Portion (g)** | **Calories** | **Carbohydrates (g)** | **Fats (g)** | **Proteins (g)** |
| --- | --- | --- | --- | --- | --- | --- |
| 8 oz Cheeseburger | 1 cheeseburger (~227g) | 227 | 550 | 36 | 50 | 34 |
| Small 8" Cheese Pizza | 1 small pie (~576g) | 576 | 850 | 94 | 36 | 38 |
| Chicken Finger Platter | 5 chicken fingers (~300g) | 100 | 299 | 16 | 18 | 18 |
| Side French Fries | 1 container (~400g) | 100 | 292 | 37 | 15 | 3.7 |
| Grilled Chicken Wrap | 1 wrap (~433g) | 433 | 600 | 68 | 14 | 54 |
| Garden Salad (with egg) | 1 container (~500g) | 220 | 161 | 15 | 11 | 2.3 |
| Side Grilled Chicken | 1 container (~450g) | 100 | 165 | 0 | 3.6 | 31 |
| Tofu cubes | 1 bag (~500g) | 81 | 117 | 2.2 | 7 | 14 |
| Kraft Viva Italian Dressing ◊ | 1 bottle (16floz) | 30 | 90 | 3 | 9 | 0 |
| Hidden Valley Original Ranch Dressing ◊ | 1 bottle (16floz) | 30 | 140 | 2 | 14 | 0 |
| Hellmann’s Mayonnaise ◊ | 1 bottle (11.5 floz) | 14 | 100 | 0 | 11 | 0 |
| Heinz Relish ◊ | 1 bottle(12.7floz) | 15 | 15 | 3 | 0 | 0 |
| Heinz Yellow Mustard | 1 bottle (14floz) | 5 | 0 | 0 | 0 | 0 |
| Heinz Ketchup | 1 bottle (32floz) | 17 | 20 | 5 | 0 | 0 |
| Kraft Mac n Cheese Single Serve Cup | 1 cup | 58 | 220 | 39 | 3.5 | 7 |
| Baby Carrots | 12 carrots | 85 | 35 | 8 | 0 | 1 |
| Celery Sticks | 12 celery sticks | 28 | 15 | 3 | 0 | 1 |
| Sabra Original Hummus | 5 oz container | 28 | 70 | 4 | 5 | 2 |
| Quaker’s Rice Cakes | 3 rice cakes | 10 | 40 | 8 | 0 | 1 |
| Jiff Creamy Peanut Butter | 5 oz container | 33 | 190 | 8 | 16 | 7 |
| Justin’s Creamy Almond Butter | 5 oz container | 32 | 190 | 8 | 16 | 6 |
| Apples | 1 honeycrisp, 1 granny smith | 100 | 52 | 14 | 0.2 | 0.3 |
| Blueberries | 12 oz cup | 100 | 57 | 14 | 0.3 | 0.7 |
| Strawberries | 12 oz cup | 100 | 33 | 8 | 0.3 | 0.7 |
| Bananas | 1 banana | 100 | 89 | 23 | 0.3 | 1.1 |
| Siggi’s Whole fat Yogurt | 1 vanilla, 1 strawberry cup | 150 | 140 | 20 | 4.5 | 5 |
| Siggi’s Lite Yogurt | 1 vanilla, 1 strawberry cup | 150 | 70 | 13 | 0 | 5 |
| Quaker Simply Granola | 5 oz container | 51 | 210 | 38 | 5 | 5 |
| Tasty Cake Powdered Donuts | 6 powdered donuts | 57 | 270 | 29 | 16 | 3 |
| Little Debbie Cosmic Brownies | 3 cosmic brownies | 66 | 280 | 42 | 11 | 2 |
| Chips Ahoy Chocolate Chip Cookies | 5 chocolate chip cookies | 33 | 160 | 22 | 8 | 1 |
| 90% Lindt Cocoa Dark Chocolate | 3 squares | 30 | 200 | 9 | 16 | 4 |
| Lay’s Potato Chips | 10 oz bag | 28 | 160 | 15 | 10 | 2 |
| Minute Maid Apple Juice | 10 oz bottle | 10oz | 140 | 34 | 0 | 0 |
| Rosenburger’s Whole Milk | 16 oz bottle | 16oz | 160 | 13 | 8 | 9 |
| Diet Coke | 12 oz can | 12oz | 0 | 0 | 0 | 0 |
| Sprite | 12 oz can | 12oz | 140 | 38 | 0 | 0 |
| **Alternative items for special diets** | |  |  |  |  |  |
| Veggie Burger* | 1 veggie burger (~227g) | 227 | 400 | 42 | 15 | 22 |
| Mozzarella Sticks* | 6 mozzarella sticks (~260g) | 86 | 440 | 37 | 23 | 19 |
| Greek Veggie Wrap* | 1 wrap (~400g) | 400 | 460 | 63 | 18 | 13 |

Supplemental Table 2. *Buffet Challenge Variable Descriptions*

| **Buffet Challenge Variable** | **Variable Description** |
| --- | --- |
| **Behavioral latency (seconds)** | **The following variables are the duration of time until a behavior begins. Time begins after research staff finishes all instructions at the beginning of the challenge, leaves and closes the door.** |
| Approach serving table | Time until participant reaches edge of buffet table. |
| Eating | Time until participant initiates first bite. |
| Nibbling/picking | Time until nibbling/picking behavior (see below) begins. |
| Serve Food | Time until participant puts first food item on plate or picks up first food item (e.g. banana) from serving table. |
| **Behavior frequency** | **The following behaviors are a frequency count, i.e., how may times a participant engaged in a behavior.** |
| Approach serving table | Approaches the buffet and takes at least one food item, with any participant serving food scoring at least 1. |
| Dissecting | Removing parts of food from the whole and discarding (e.g., removing chicken from wrap, taking cheese off pizza, etc.) |
| Dropping/hiding food | Any attempt to remove food from plate. This includes attempts at hiding food mixed in with trash, dropping food onto the floor, etc. |
| Eating whilst serving | Any instance of participant eating at the serving table. Each bite is one occurrence. |
| Time Spent Serving Food | Time is measured from when the adolescent picks up his/her first serving utensil or food item at the buffet and is concluded when the adolescent puts down his/her last serving utensil or grabs last food item. Only refers to initial serve/trip to buffet table. |
| Hand fidgeting | Non-eating related movements of the hands: tapping, twiddling, twirling hair around finger, inspecting hair, snapping, etc. |
| Inappropriate utensil use | Using utensils for traditionally handheld foods (e.g., cheeseburger). |
| Label checking | Any attempt to look at a nutritional label on food item. Each time a label is checked is an occurrence. |
| Napkin use | Any occurrence of using a napkin or clothing to wipe hands or mouth. Also spitting into napkin. |
| Nibbling/picking | Each occurrence of participant taking small bite of food (not using utensils) or picking off a small piece of food to eat (e.g., one candy off a Cosmic Brownie). Bites should be more than one inch squared approximately. |
| Smearing food | Any attempt at spreading out or flattening food on the plate. |
| Staring at food | Gaze is fixed on food (either food they have served themselves or food on the buffet table) and not engaged in any other behaviors (including chewing). Participant must be staring >=6 seconds to qualify as a stare. |
| Tearing food | Ripping food into pieces or crumpling food. |
| **Behavior duration (secs)** | **The following variables are timed from the initiation of a given behavior until its final completion.** |
| Eating | Time elapsed from when the first bite is taken to when the last bite is taken. |
| Serving | Time elapsed from participant picking up the first food to leaving the serving table. This includes each time participant reapproaches buffet table to serve. |
| Staring at food | Duration of gaze fixed on food, with other behaviors for <5 seconds. Cumulative for multiple instances of staring. |

*Supplemental Figure 1.*
Buffet Challenge Layout


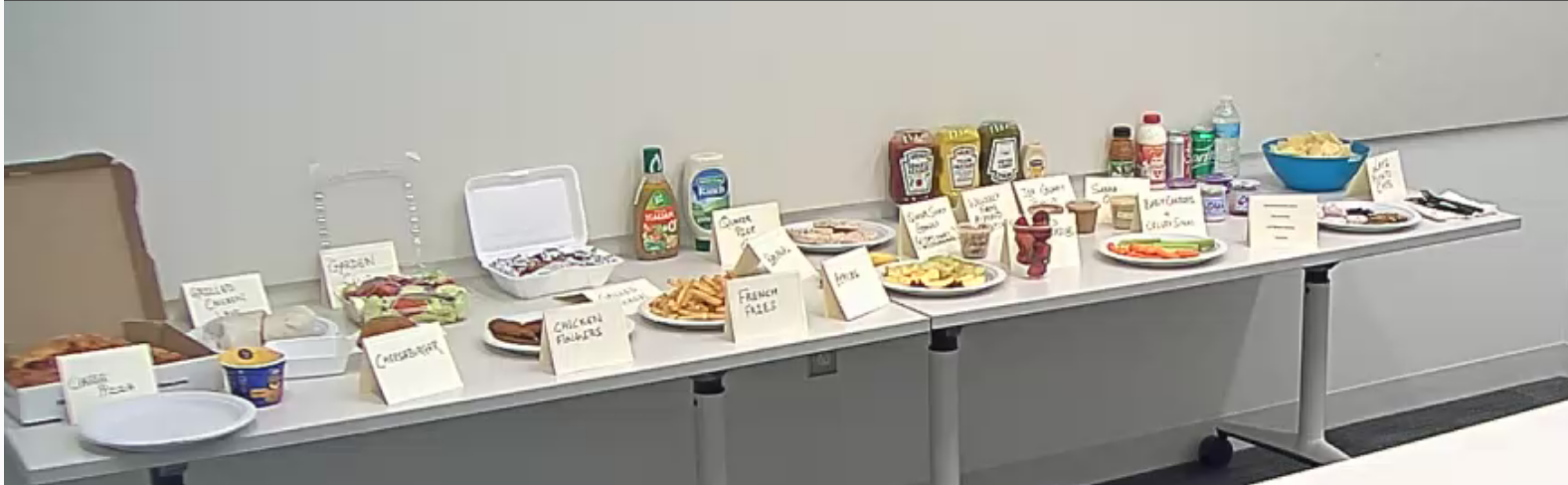

Supplement: Supplementary file 1 — Additional file 1. Supplemental information. [file 40337_2024_968_MOESM1_ESM.docx]
